# Fructose Intake, Serum Uric Acid, and Cardiometabolic Disorders: A Critical Review

**DOI:** 10.3390/nu9040395

**Published:** 2017-04-18

**Authors:** Cristiana Caliceti, Donato Calabria, Aldo Roda, Arrigo F. G. Cicero

**Affiliations:** 1 Department of Chemistry “Giacomo Ciamician”, Alma Mater Studiorum, University of Bologna, 40126 Bologna, Italy; cristiana.caliceti@unibo.it (C.C.); aldo.roda@unibo.it (A.R.); 2 Istituto Nazionale Biostrutture e Biosistemi (INBB), 00136 Rome, Italy; 3 Centro Interdipartimentale di Ricerca Industriale Energia e Ambiente (CIRI EA), Alma Mater Studiorum, University of Bologna, 47900 Rimini, Italy; donato.calabria2@unibo.it; 4 Department of Medical and Surgical Sciences, Alma Mater Studiorum, University of Bologna, 40138 Bologna, Italy

**Keywords:** fructose, uric acid, cardiometabolic disorders, xanthine oxidase, pathophysiology, epidemiology

## Abstract

There is a direct relationship between fructose intake and serum levels of uric acid (UA), which is the final product of purine metabolism. Recent preclinical and clinical evidence suggests that chronic hyperuricemia is an independent risk factor for hypertension, metabolic syndrome, and cardiovascular disease. It is probably also an independent risk factor for chronic kidney disease, Type 2 diabetes, and cognitive decline. These relationships have been observed for high serum UA levels (>5.5 mg/dL in women and >6 mg/dL in men), but also for normal to high serum UA levels (5–6 mg/dL). In this regard, blood UA levels are much higher in industrialized countries than in the rest of the world. Xanthine-oxidase inhibitors can reduce UA and seem to minimize its negative effects on vascular health. Other dietary and pathophysiological factors are also related to UA production. However, the role of fructose-derived UA in the pathogenesis of cardiometabolic disorders has not yet been fully clarified. Here, we critically review recent research on the biochemistry of UA production, the relationship between fructose intake and UA production, and how this relationship is linked to cardiometabolic disorders.

## 1. Introduction

Uric acid (UA) is the final product of purine metabolism. It is a well-known risk factor for gout [1]. Moreover, a growing body of evidence suggests that high levels of serum UA are also biomarkers for cardiovascular disease (CVD) morbidity and mortality [2].

The increased incidence of gout among rich people in the 18th and 19th centuries was principally due to high consumption of purine-containing meat. However, UA levels are rising in the 21st century too, with mean levels of >5.5 mg/dL in women and >6.0 mg/dL in men [3]. This can partly be explained by a remarkable increase in added sugars in the Western diet, especially fructose [4,5,6]. Accordingly, blood UA levels are higher in Western countries than in the rest of the world. In non-Western countries, hyperuricemia is relatively rare in rural communities. However, there is increased migration from rural areas to cities or communities where the Western diet is dominant and hyperuricemia is more prevalent [7].

Recent research suggests that hyperuricemia may be caused by elevated activity of the enzyme xanthine oxidase (XO) [8]. Xanthine oxidase inhibitors (XOI) have thus been proposed as a strategy for reducing UA and oxidative stress. Both are risk factors for gout, chronic kidney disease (CKD), CVD, obesity, insulin resistance, and metabolic syndrome. Humans and great apes produce UA via XO-catalyzed oxidation of purines. Unlike other mammals, humans and great apes cannot synthesize the uricase enzyme (urate oxidase) and so cannot metabolize UA to allantoin. As a result, UA blood concentrations in humans and great apes are at least 10 times higher than in other mammals, with the consequent risk of developing hyperuricemia [9]. 

High UA levels favor adipose tissue formation, which was originally an evolutionary advantage for humans [7]. Nowadays, however, excess adipose tissue is considered a predisposing factor for insulin resistance, obesity, and hypertension [10]. This excessive fat storage may be due to increased consumption of fructose-enriched food and drink, which raises serum UA levels [11]. Indeed, several clinical studies have shown that the administration of allopurinol, a competitive antagonist of XO, can significantly improve endothelial function and the circulating markers of oxidative stress in patients with, or at risk of, CVD [12].

Here, we review the most relevant discoveries in the field, focusing on (i) the role of UA in cardiometabolic disorders; and (ii) the link between fructose consumption, high blood UA levels, and associated disorders, particularly CVD. 

## 2. Search Strategy (Methods)

We conducted a literature search of different scientific databases (including Scopus, Google Scholar, PubMed, and Web of Science) for peer-reviewed studies focusing on XO, hyperuricemia, fructose, and CVD. The search strategy was designed to retrieve studies published in English from journal inception to 2016. We used an assessment framework to appraise the quality of basic research studies, prognostic studies, and methodological considerations in the analysis and publication of observational studies. The screening, study selection, and data extraction was undertaken by three independent authors. Disagreement was resolved by discussion and, if required, by a fourth independent author. We have assessed the clinical and methodological heterogeneity across the studies and, where available, included meta-analyses whenever these have been performed. 

## 3. Purine Metabolism and Uric Acid Physiology

**Figure 1:**
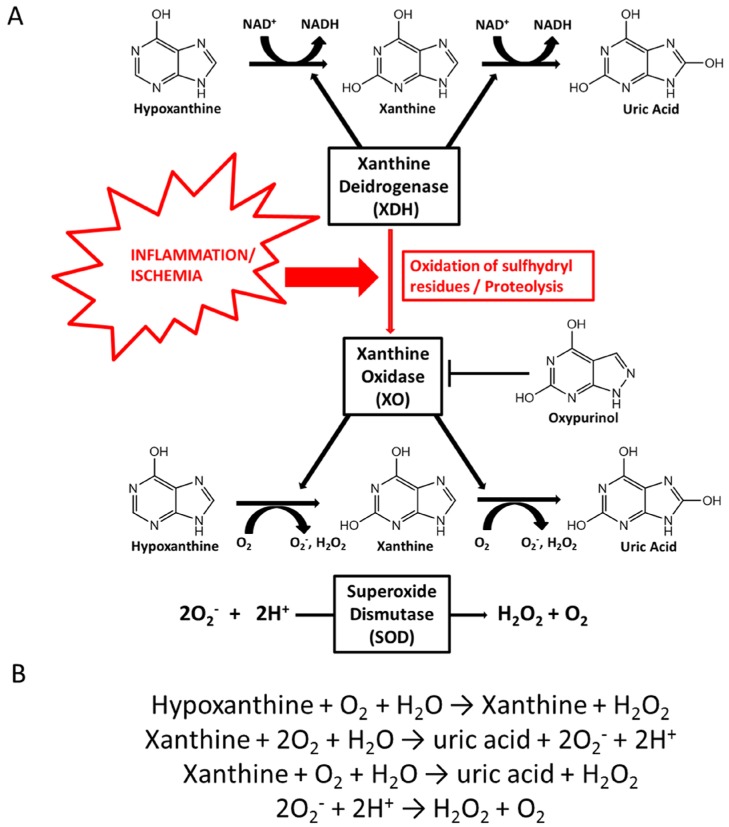
Uric acid formation through xanthine oxidase activity. (**A**) Under ischemic or inflammatory conditions, xanthine dehydrogenase (XDH) is converted to xanthine oxidase (XO) via the oxidation of sulfhydryl residues or proteolysis of XDH. In the presence of oxygen, XO catalyses the oxidation of hypoxanthine to xanthine and then to uric acid (UA), with consequent production of the superoxide anion (O_2_^−^) and hydrogen peroxide (H_2_O_2_). The competitive antagonist allopurinol is converted in the active form, oxypurinol, via XO activity, acting as an XO inhibitor; (**B**) During hypoxanthine conversion to xanthine and then UA, high levels of H_2_O_2_ and O_2_^−^ are produced and converted to O_2_ and H_2_O_2_, spontaneously or in a reaction catalyzed by the enzyme superoxide dismutase (SOD).

Purines are generated through two pathways. First, there is de novo synthesis from non-purine compounds, such as amino acids and bicarbonate, regulated by phosphoribosyl-pyrophosphate synthetase (PRPP). Second, there is the purine salvage pathway, which economizes the intracellular energy expenditure and is regulated by hypoxanthine-guanine phosphoribosyltransferase (HG-PRTase) [13]. Catabolism of purines is regulated by xanthine-oxidoreductase (XOR), coding for two distinct enzymatic forms: xanthine dehydrogenase (XDH) and XO [14]. XDH and XO catalyze the oxidation of hypoxanthine to xanthine and subsequently to UA, which is the hepatic and intestinal metabolite of purine [15,16]. The main difference between XDH and XO is that XDH-FAD reacts predominantly with NAD^+^, whereas XO-FAD has higher reactivity for molecular oxygen, producing higher levels of hydrogen peroxide (H_2_O_2_) and the superoxide anion (O_2_^−^). O_2_^−^ is then converted into oxygen and H_2_O_2_, either spontaneously or catalyzed by the superoxide dismutase (SOD) enzyme, (Figure 1A,B) [14]. XO is the form that is most abundant in inflamed vascular and ischemic tissues. XDH can be converted into XO via the oxidation of sulfhydryl residues or the proteolysis of XDH (Figure 1A) [17]. Allopurinol and its active metabolites oxypurinol act as competitive antagonists of XO and can lower UA levels. In addition, allopurinol has consistently been reported to prevent H_2_O_2_ production [12,18] (Figure 1B). 

Most UA is filtered by the kidneys and eliminated in urine. The rest passes through the gut and is cleaved by bacteria into waste substances, which are eliminated in faeces. Inefficient renal excretion of UA is the main cause of both primary and secondary hyperuricemia [19]. Renal UA excretion is regulated by several transporters. Renal UA reabsorption is mediated by urate transporter 1 (URAT1, also known as SLC22A12) and glucose transporter 9 (GLUT9, also known as SLC2A9) [20,21]. Renal UA secretion is mediated by sodium phosphate transporter (NPT1, also known as SLC17A1) [22,23]. UA concentration can be decreased by several drugs (benzbromarone, losartan, probenecid, sulfinpyrazon). These drugs mostly work by URAT1 inhibition, which explains their uricosuric effect [24]. UA excretion is also regulated by breast cancer resistance protein (BCRP, also known as ABCG2), which belongs to the superfamily of ATP-binding cassette (ABC) transporters [25,26,27]. Unlike ABC transporters, however, BCRP has only one N-terminal ATP-binding domain [28,29,30]. Reduced intestinal excretion of UA is often associated with polymorphisms of the BCRP gene [26] or with a lack of BCRP dimerization, which is due to oxidative stress [31,32].

As noted above, most mammals can degrade UA to allantoin in a reaction catalyzed by uricase, an enzyme present in peroxisomes. Allantoin is subsequently degraded to urea for excretion [33,34]. Most mammals thus have relatively low UA circulation levels (0.5 to 2.0 mg/dL). However, humans and great apes cannot synthesize functional uricase and therefore have much higher blood UA levels.

According to Neel’s hypothesis, our closest evolutionary ancestors underwent functional genetic mutations, which silenced genes involved in the degradation of UA and the synthesis of vitamin C [10], which is the major water-soluble intracellular free-radical scavenger in human plasma [35]. These mutations increased de novo lipogenesis and weight gain [36]. This is because vitamin C competes with UA for renal resorption in the proximal tubule [37,38,39], producing a uricosuric effect. UA causes endoplasmic reticulum stress; this, in turn, activates SREBP-1c, which stimulates fat accumulation in the liver [40]. Indeed, Cheung et al. recently reported that XOR-knockout mice have a central defect in adipogenesis and fail to gain fat [41,42,43]. Thus, there is a positive correlation between circulating UA and obesity, especially visceral obesity [44]. Accordingly, although hyperuricemia is often considered to be a secondary phenomenon in metabolic syndrome, it is also an independent predictive factor for obesity and hyperinsulinemia [4]. In themselves, UA accumulation and lack of vitamin C do not cause obesity. Rather, they increase susceptibility to obesity and diabetes as a result of an interaction between genetic factors (mostly a polygenic contribution) and environmental factors such as lifestyle, social influences, and fetal surroundings [11,45]. 

## 4. Fructose Metabolism and the Mechanisms by Which Fructose May Contribute to Uric Acid Production 

In the past 100 years, there has been a progressive rise in blood UA levels, especially in Western countries. This is associated, at least in part, with a rise in the number of people consuming a Western diet. In particular, there has been increased consumption of fructose-containing sugars [46,47], sucrose, high-fructose corn syrup, soft drinks, and fruit juices [11,48]. 

Fructose is present as a monosaccharide in fruits and vegetables [49], as a disaccharide in sucrose (with d-glucose), and as oligo- and polysaccharides (fructans) in many plants. It is also used as an added sweetener for food and drink, and as an excipient in pharmaceutical preparations, syrups, and solutions [50]. Following fructose ingestion, blood glucose levels are lower (GI 32) than after ingesting a similar amount of glucose (GI 100) or sucrose (GI 68). Similarly, insulin levels do not increase significantly after fructose ingestion. Moreover, fructose has a greater sweetening power than sucrose, so smaller quantities of fructose can be used to sweeten foods. Finally, its calorific value is 3.75 kcal/g, slightly lower than that of sucrose (3.92 kcal/g) [51]. It is not yet clear if a moderate intake of fructose significantly promotes the formation of advanced glycation products (AGE), which damage tissues and thus contribute to ageing and metabolic disorders [52]. Several studies suggest that a fructose-rich diet has negative metabolic consequences, including AGE formation [52,53,54]. However, the effect of a long-term fructose intake on AGE accumulation in tissues has not yet been studied in healthy volunteers.

**Figure 2:**
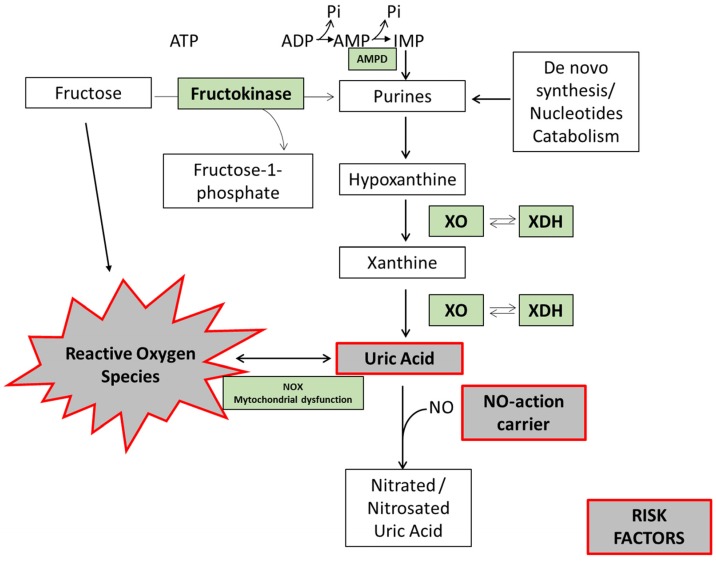
Fructose-induced uric acid formation: risk factor biomarkers. In hepatocytes, fructokinase catalyzes the rapid phosphorylation of fructose to fructose-1-phosphate, using ATP as a phosphate donor. Intracellular phosphate (Pi) levels decrease, stimulating the activity of AMP deaminase (AMPD). AMPD converts AMP to inosine monophosphate (IMP). IMP is metabolized to inosine, which is further degraded to xanthine and hypoxanthine by xanthine oxidase (XO), ultimately generating uric acid (UA). UA can react with nitric oxide (NO), reducing NO bioavailability and inducing dinucleotide phosphate oxidase (NOX) activation and mitochondrial dysfunction. In turn, this promotes oxidative stress and endothelial dysfunction. Fructose per se can also induce oxidative stress.

Hyperuricemia is caused by the overproduction and/or underexcretion of UA. It has been reported that metabolism of fructose stimulates UA production, since transient ATP depletion commonly occurs with the generation of AMP [55] and reduced UA excretion [56]. Briefly, during fructose metabolism, fructose is phosphorylated into fructose 1-phosphate in a reaction catalyzed by fructokinase primarily in the liver. This reaction is rapid, has no negative feedback, and hugely decreases the levels of intracellular phosphate and ATP [57]. Next, the enzyme fructose-1-p aldolase (also known as aldolase B) breaks fructose 1-phosphate into dihydroxyacetone phosphate (DHAP) and d-glyceraldehyde. When there is a high intake of fructose, phosphorylation into fructose 1-phosphate is fast, but the reaction with aldolase is slow (Figure 2). Hence, fructose 1-phosphate accumulates, and intracellular phosphate decreases. This decrease stimulates AMP deaminase (AMPD), which catalyzes the degradation of AMP to inosine monophosphate, increasing the rate of purine degradation [58] (Figure 2). The purine degradation produces UA [59] and generates mitochondrial oxidants [36] (Figure 2). Mitochondrial oxidative stress then induces aconitase inhibition in the Krebs cycle, with accumulation of citrate and stimulation of ATP citrate lyase and fatty acid synthase. The result is de novo lipogenesis and hepatic fat accumulation [36]. 

Physiologically, the increase in intracellular UA is followed by an acute rise in circulating levels of UA, which is likely due to its release from the liver [57,60]. Fructose also stimulates UA synthesis from amino acid precursors such as glycine [61]. Moreover, long-term fructose administration suppresses renal excretion of UA, resulting in elevated serum UA levels [19]. Kaneko and colleagues found that a single administration of fructose affects the excretion of UA to the intestinal lumen, inducing the suppression of BCRP dimerization by reactive oxygen species (ROS)-derived production of dinucleotide phosphate (NADPH) oxidase (NOX) [56]. 

## 5. Mechanisms by Which Fructose-Uric Acid May Contribute to Cardiometabolic Disorders

UA has been described as a “paradox molecule” with opposing roles. At physiological concentrations (much higher than ascorbate concentrations in plasma), it is a powerful oxygen radical scavenger in extracellular hydrophilic environments such as human plasma. It may protect the erythrocyte membrane from lipid peroxidation [35]. UA can react with O_2_^−^, H_2_O_2_, hydroxyl radical (OH^−^), and particularly peroxynitrite (OONO^−^) [35,62,63]. However, several authors suggest that UA also acts as a prooxidant inside the cell under certain inflammatory conditions, such as atheromatous plaque formation [35,64]. Recent studies have shown that UA can induce intracellular oxidative stress and proinflammatory effects in various cell types [65,66]. It does this by stimulating NOX [67,68] and by altering mitochondrial function with the consequent alteration in fat synthesis [36]. Fructose also induces NOX activation [69,70]. 

Oxidative stress significantly contributes to the development of insulin resistance and imbalance in vascular homeostasis, including endothelial cell dysfunction, atherosclerosis, vascular calcification, and impaired myocardial energetics, stimulating the production of interleukin-1 (IL-1), interleukin-6 (IL-6) and tumor necrosis factor α (TNFα) [71,72]. Heme oxygenase 1 (HO1), a potent antioxidant, decreases UA levels and adipocyte dysfunction by decreasing levels of ROS and XO [73]. XO is one of the major endothelial sources of O_2_^−^ and H_2_O_2_. XO levels are substantially elevated in patients with coronary disease or carotid stenosis, and there is an inverse relationship between XO levels and endothelium-dependent vasodilation [74]. Accordingly, UA inhibits the bioavailability of nitric oxide (NO), which is a vasodilator [65,75]. Extracellular UA can also enter endothelial cells and vascular smooth muscle cells through URAT1, GLUT9, and potentially other transporters [76,77] activating the NF-κB axis, which leads to an increase in MCP-1, IL-8, VCAM-1, and ICAM-1 [78]. As a result, it has been hypothesized that intracellular XO activity and increased ROS production might be factors in endothelial dysfunction, which leads to the development of essential hypertension [79]. In this regard, ROS generation and vascular endothelial dysfunction can be reduced by drugs such as allopurinol and febuxostat, which inhibit XO activity and consequently reduce UA production [80].

OONO^−^ is a potent non-radical oxidant species formed by the reaction between NO and O_2_^−^, which commonly occurs in the vascular endothelium [81]. NO is usually generated by NO synthase (NOS), However, under hypoxic conditions, XO can also modulate NO concentrations [82,83]. Since reducing NO bioavailability induces endothelial dysfunction and oxidative stress, XO inhibition could prevent oxidative damage, at least in part, by restoring NO generation [84,85]. Several studies have shown that, under aerobic conditions, UA can react with NO but not with OONO^−^, producing a nitrosated derivative [35,86,87]. Therefore, despite the general belief that survival is associated with increased antioxidant capacity, the opposite appears to be true: the ability to increase oxidative stress may have been associated with survival among early hominoids. 

## 6. Evidence from Clinical Studies, Relevance for Humans

Physiologically, circulating UA levels increase with age. UA levels are lower in women of childbearing age, rising after menopause to levels similar to those in men [24]. There is still no clear threshold, above which uricemia becomes “abnormal”. The pathophysiological approach uses the supersaturation concentration of UA, 6.8 mg/dL at 37 °C, as a cut-off value, as indicated by guidelines for gout management [88]. However, recent findings suggest that this should be revised with reference to rheumatic, cardiovascular, and renal risk. Hyperuricemia is increasing in prevalence, as are its associated pathological conditions, such as metabolic syndrome, CKD, and CVD [8,89]. The average serum UA levels in the general population are rising [90] due to dietary changes, rising body mass indexes (BMI), and improved life expectancy. 

Several in vitro and in vivo studies have shown that high fructose consumption increases blood UA levels [4,55,56,91]. In addition, consuming fructose over several days [70] and intravenous fructose administration [92] are both associated with an increase in fasting serum UA levels. It is not yet clear whether circulating UA levels increase when fructose is taken in small doses over several days or when it is taken as a single large dose. It is also unclear whether a high-fructose diet (HFrD) alters the renal clearance of UA or the fractional excretion of UA (UAFE) [93].

A relatively old meta-analysis of 21 controlled feeding trials of at least seven days showed that isocaloric exchange of fructose for other carbohydrates did not affect serum UA. However, in nondiabetic participants, hypercaloric supplementation of control diets with fructose (+35% excess energy) at extreme doses (213–219 g/day) significantly increased serum UA compared with the control diets alone (MD = 31.0 mmol/L (95% CI: 15.4, 46.5)) [94]. This meta-analysis was conducted before the publication of more recent trials, which confirmed the relationship between fructose intake and serum UA levels [95,96,97]. Thus, the available evidence is yet partly conflicting. 

Notably, the fructose-induced increase in serum UA may cause acute damage (post-assumption). By excluding shorter studies, the meta-analysis may have excluded insights into this issue [97]. Finally, a fructose-restricted diet has been associated with a decrease in serum UA [98].

Lecoultre and colleagues performed a study on healthy subjects with two approaches: four to six days on an isoenergetic low-fructose diet (LFrD) and then either 6 days on a hyperenergetic HFrD with 34% excess energy as fructose or four days on a weight-maintenance HFrD in which 30% starch was substituted with fructose [42]. There was no difference in urinary UA excretion after the HFrD and the LFrD [42]. This suggests that UA production increases mainly when there is abnormally high hepatic metabolism of fructose. These findings also demonstrate that decreased urinary UA excretion may contribute to fructose-induced hyperuricemia. This mechanism could substantially increase the risk of gout in people who consume high amounts of fructose. Moreover, chronic exposure to fructose favours the onset of metabolic syndrome and increases insulin resistance [51].

Many experiments have shown that hyperuricemia may have a potential role in endothelial dysfunction and reduced NO bioavailability. However, several other studies have failed to demonstrate that an increase in blood UA levels can contribute to the onset of coronary and cardiovascular diseases. These studies also found no association between raised serum UA levels and the incidence of cardiovascular events [35,99,100,101,102,103]. It is not yet possible to say whether UA is a causal, compensatory, or coincidental factor for CVD [104]. Generally, gout and hyperuricemia patients also suffer from hypertension, CKD, insulin resistance, and obesity [105]. However, no clear link has yet been demonstrated between a HFrD and CVD. A recent systematic review and meta-analysis of prospective cohort studies was undertaken to quantify the association between intake of fructose-containing sugar (high-fructose corn syrup, sucrose, and fructose) and incidence of hypertension. This review searched MEDLINE, EMBASE, CINAHL, and the Cochrane Library for relevant studies [106]. It demonstrated that there was no association between a high total fructose intake and an increased risk of hypertension in three large prospective cohorts of men and women in the USA. In contrast, the same group conducted another systematic review and meta-analysis of prospective cohort studies to quantify the association between the consumption of fructose-containing sugar-sweetened beverages (SSBs) and the risk of hypertension. This demonstrated that SSBs were associated with a modest risk of developing hypertension in six cohorts [107]. 

The well-characterized Mediterranean cohort of the Brisighella Heart Study has a high intake of fruit [108]. Here, the increase in blood UA levels in the general population has been associated with increased incidence of hypertension and diabetes [109], LDL oxidation [110], arterial stiffness [111], impaired cognitive function [112], and heart failure [113]. 

In general, epidemiologists and other scientists have attempted to prove a causal link between dietary fructose intake and metabolic disorders, such as obesity, diabetes, and metabolic syndrome. However, while some studies have found a link, other studies have not [114].

Recent relevant clinical research has sought to verify if XO inhibition could benefit patients with high circulating UA levels, focusing on the XO inhibitor allopurinol and its active metabolite oxypurinol. Allopurinol has displayed beneficial effects on blood pressure in adolescents with newly diagnosed essential hypertension [89], and in patients with heart failure, coronary artery disease, and stroke [115]. Allopurinol may thus improve endothelial function and endothelium-dependent vasodilation in chronic heart failure patients [18].

XO inhibition has also been shown to improve a range of surrogate markers in hyperuricemic patients with CVD [9]. Wu and colleagues have demonstrated that high blood UA levels could be an independent predictor of mortality in patients with severe heart failure [116]. Baldus and colleagues have shown that oxypurinol improves myocardial contractility in patients with ischemic cardiomyopathy [117]. It also improves coronary vascular endothelial dysfunction in patients with coronary artery disease (CAD) [74]. The same group has shown that, without XO inhibition, lowering UA by uricosuric treatment (with benzbromarone) has no beneficial effect for chronic heart failure (CHF) patients [118]. This finding suggests that it is upregulated XO activity, rather than increased UA, that is actively involved in hemodynamic impairment in CHF. However, other studies suggest that elevated serum UA levels may be a risk marker for developing CVD [119,120]. Therefore, the scientific community needs to clarify the role of XO activity in CVD, so that the administration of natural or synthetic XO inhibitors as therapeutic agents for CVD can be properly evaluated [121,122].

## 7. Conclusions

In summary, a specific causal link between fructose consumption, hyperuricemia, and CVD has not yet been established. There is an association between UA and established cardiovascular risk factors, and there is a limit to how much population-based studies can adjust for confounding variables. As such, it is not yet possible to conclude that fructose intake is the main contributor to an increase in blood UA, and that this detrimentally affects vascular health. Further studies are required to prove or exclude a causal correlation between dietary fructose intake, UA production, and metabolic disorders. This would allow researchers to better understand which patients would obtain the greatest preventative benefit from reducing their UA levels, with diet and/or with XO inhibitors. 

An important issue to consider is the bioavailability of XO inhibitors, especially the ability to cross cell plasma membranes. To address this, members of our group recently developed a cell-based biosensor that only measures intracellular XO activity and its inhibition by drugs that cross cell membranes [123]. The scientific community will need to focus on bioanalytical methods for directly monitoring XO activity and UA production. These will be useful tools for predicting the potential effects of new XO inhibitors, which could be used to treat hyperuricemia linked with cardiometabolic disorders.
